# Therapy Changes During Pemphigus Management: A Retrospective Analysis

**DOI:** 10.3389/fmed.2020.581820

**Published:** 2020-11-25

**Authors:** Roberta Scarpone, Wojciech Francuzik, Margitta Worm, Guido Heine

**Affiliations:** ^1^Division of Allergy and Immunology, Department of Dermatology, Venereology and Allergy, Charité – Universitätsmedizin Berlin, Berlin, Germany; ^2^Division of Allergy, Department of Dermatology and Allergy, University Hospital Schleswig-Holstein, Kiel, Germany

**Keywords:** pemphigus, treatment, therapy changes, retrospective study, autoantibodies

## Abstract

Pemphigus diseases are rare, and the treatment response differs between patients. Several therapy changes are often required to achieve disease control and avoid unwanted side effects. We aimed to analyze the treatment courses of pemphigus patients and the clinical responses regarding therapy changes. Pemphigus patients in our center were retrospectively examined according to the medication and dosage, disease activity, reason for treatment changes, and autoantibody concentrations. Therapy changes due to insufficient therapeutic effects or side effects were analyzed. Seventy-seven pemphigus patients with repeated consultations were identified (81% pemphigus vulgaris, 19% pemphigus foliaceus). Disease control was achieved in 66 patients (86%; score “almost clear” or “clear”), with an average of 4 different therapy regimens (range 1–18 changes), after an average of 2 years of treatment (range 0–11 years). Twenty-two patients (29%) with refractory disease received rituximab, of which 19 (86%) subsequently achieved remission. Anti-desmoglein-1 and−3 concentrations correlated with disease severity, but not with the number of treatment changes. The identification of an effective and safe therapy for the individual pemphigus patient is a challenge and often requires time, which is reflected by a high number of therapy changes. Predictive parameters are warranted to directly identify the safest and most efficient treatment regimen for an individual patient.

## Introduction

Pemphigus diseases are rare and the incidence varies around the world (1). Due to the low prevalence of the disease and variability of triggers and manifestations, large scale study data on treatment responses are limited (2–4). Currently, pemphigus therapy guidelines are based on a limited number of placebo-controlled trials (3, 5–7). The treatment response can vary among individual pemphigus patients and not rarely several therapy changes are necessary to achieve long-lasting disease control (8, 9), i.e., the absence of symptoms. However, inadequate disease control as well as iatrogenic immunosuppression can lead to infectious and other potentially fatal complications (10–12).

We investigated the treatment courses in a large pemphigus population regarding therapy changes. We assumed that a safe and efficient regimen should result in few therapy changes, while many changes indicate a difficult to treat patient.

## Methods

The medical records of pemphigus patients in our department between January 2013 and December 2018 were analyzed. The analysis was approved by the institutional ethics committee (EA4/173/18). Patients with pemphigus vulgaris or foliaceus were identified according to the ICD-10 codes L10.x and selected for further analysis if at least two presentations in our department were documented. The disease activity was retrospectively scored using a Physician Global Assessment (PGA) score [0 = clear, 1 = almost clear (post-inflammatory marks, erythema), 2 = mild (crusts, single erosions), 3 = moderate (extended erosions, blisters), and 4 = severe (spread erosions, multiple blisters, reduced general condition)]. The oral maintenance therapies were classified according to the drugs into prednisolone (2.5–30 mg), azathioprine (25–300 mg), dapsone (25–200 mg), methotrexate (7.5–25 mg), and mycophenolate mofetil (250–2,000 mg). Previous medication was continued without a specific statement unless an unwanted reaction occurred. Short-term intensified therapies, used as an initial disease control-induction regimen or for flare control during the disease course, were as follows: oral prednisolone (60–100 mg declining doses within 6–10 days, repeated 3 times in monthly intervals) or i.v. pulse therapies were methylprednisolone i.v. (3x 250 mg in 3 days and repeated ≥3 times in ≥4-weekly intervals) or cyclophosphamide/dexamethasone i.v. (500/100 mg in 3 days and repeated ≥3 times in ≥4-weekly intervals), and rituximab cycles (2x 1g within 2 weeks, repeated after 6 months unless maintained absence of symptoms and B cell depletion). The oral treatment was continued between pulse therapies and is not separately shown. Treatment changes were counted if an ongoing therapy was not efficient and the clinical symptoms did not improve, or an adverse event occurred (e.g., anemia on dapsone treatment or elevated liver enzymes or gastrointestinal disorders on azathioprine treatment). Therapies that were used less than three times between 2013 and 2018 were not included in the analysis. Autoantibody serum concentrations were determined by our routine laboratory (ELISA from Euroimmun, Germany, positive >20 U/ml according to the manufacturer's instructions). Data collection and analysis were conducted using Microsoft Excel 2016 and R. Descriptive statistical methods were applied. Data are presented as median and range.

## Results

### Patient Population

Of 981 patient visits of patients encoded ICD L10.x, 77 pemphigus patients with repeated consultations were identified, of which 62 patients (81%) were diagnosed with pemphigus vulgaris and 15 (19%) with pemphigus foliaceus (Table 1). The median age at first visit was 56 years and the median time between first symptoms and pemphigus diagnosis was 2 months (Table 1).

**Table 1:** Patient characteristics (*n* = 77).

Age, years (range)	56 (23–91)
Female/male, number (%)	44 (57)/33 (43)
Pemphigus vulgaris/foliaceus, number (%)	62 (81)/15 (19)
Body weight, kilogram (range)	80 (43–138)
First symptoms to diagnosis, months (range)	2 (0–12)
Severity score at first visit, score (number)	1 (1), 2 (12), 3 (63), 4 (1)
Time to remission, years (range)	2 (0–11)
Therapy regimens to remission, years (range)	4 (1–18)
Last remission period, years (range)	2 (0–14)

### Disease and Treatment Course During the Observation Time

**Figure 1:**
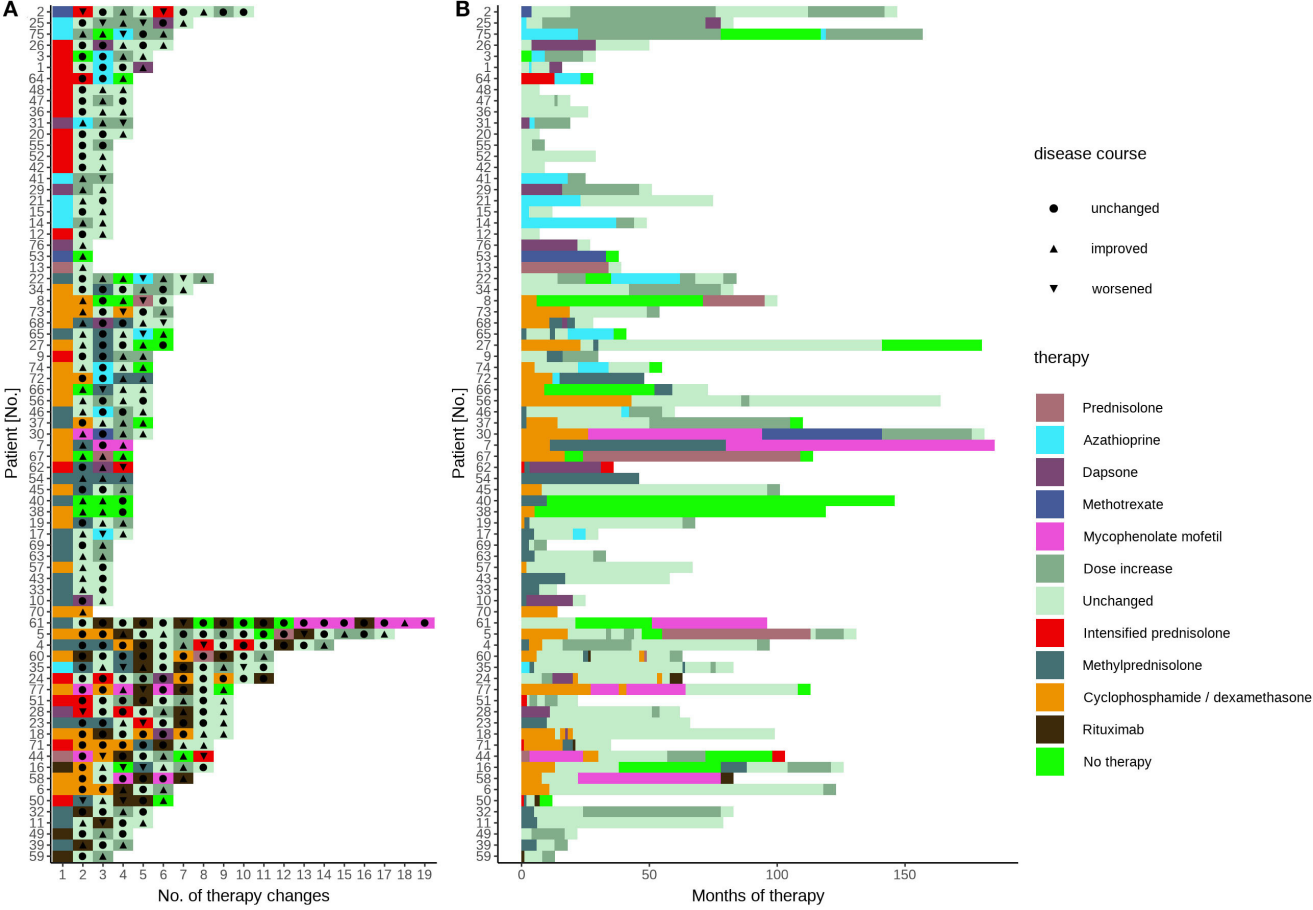
Treatment sequelae of pemphigus patients. All specific treatments of pemphigus patient's disease and treatment course in the patient cohort displayed according to **(A)** therapies grouped into oral systemic immunosuppressants (upper), pulse therapies (middle), and rituximab (lower) and **(B)** treatment duration. Each color represents the treatment after the last change or dose change. The conventional systemic therapies between pulse therapies or rituximab courses are not shown. The triangles and circles indicate clinical response.

We determined the number of different therapy regimens in our patient cohort by classifying the current drugs, dosages, clinical response, and marking each treatment change. The data show a large interindividual variation with a median of 4 therapy changes (range 1–18 changes, Figure 1A). The therapy response reflected in therapy changes was highly variable, except for rituximab (Figure 1A). In detail, after the addition of short-term intensified prednisolone over 6–10 days, eight patients showed an improvement and five patients an exacerbation. Accordingly, the symptoms in response to azathioprine improved in 25 patients and exacerbated in seven patients, as was observed for dapsone (10 and 2), methotrexate (5 and 2), and mycophenolate mofetil (3 and 2). After rituximab courses most patients improved (22 cases, 19 remissions, 2 residual activity, 1 unknown). The number of intensified oral prednisolone therapy courses for the patients that received the therapy was in median 1-time (range 1–3 times), of methylprednisolone pulse i.v. therapy in median 4 times (range 1–20), of cyclophosphamide/dexamethasone i.v. in median 9 courses (range 1–26), and of rituximab in median 1 cycle (range 1–6). The median individual observation time was 4 years (range 0–15 years, Figure 1B). The respective drug survival, defined as the time until a therapy change became necessary, showed large interindividual differences. In detail, the drug survival of prednisolone was 3 years (range 0–14 years), and accordingly for azathioprine 1 year (range 0–13 years), dapsone 1 year (range 0–6 years), methotrexate 6 years (range 0–12 years), mycophenolate mofetil 5.5 years (range 2–14 years). The analysis of the documented adverse effects showed that these were most common during azathioprine intake. In detail, seven patients (9%) had to discontinue azathioprine due to elevated liver enzymes (five patients, 6%) or gastrointestinal disorders (two patients, 3%, data not shown otherwise). Sepsis occurred in 3 initially severely affected patients (4%) who received rituximab.

Analysis of the treatment response showed in our cohort 66 patients (86%) achieving disease control, defined as a score of 1 (“almost clear”) or 0 (“clear”); see Table 1. The median required time was 2 years (range 0–11 years) and the median number of different therapy regimens to achieve disease control was 4 (range 1–18 changes, Table 1). In 11 patients (14%), remission without the need for any further systemic therapy during the investigation time was achieved. Twenty-two patients (29%) with refractory pemphigus were treated with rituximab. A disease control score of 1 or 0 was observed in the majority of those patients (19 patients). In two patients (3% overall) additional rituximab courses were required due to residual disease activity, and one patient received rituximab immediately before closing of the database. During the whole analysis period, we observed one fatal disease course, which occurred before rituximab was approved for pemphigus treatment, in a patient with a clinically severe phenotype at the initial presentation (Patient #68, initial score 4, P. foliaceus). In this case, repeated months without systemic therapy due to a lack of compliance were noticed, along with side effects from glucocorticosteroids (refractory type-II diabetes), steroid-sparing drugs (gastrointestinal complaints after azathioprine, anemia after dapsone), or pulse therapies (infections after cyclophosphamide/dexamethasone, pneumonia after methylprednisolone), followed by severe disease exacerbation. This case underlines the requirement for the selection of a safe treatment with rapid efficacy.

### Autoantibody Serum Concentrations Do Not Predict the Treatment Changes

**Figure 2:**
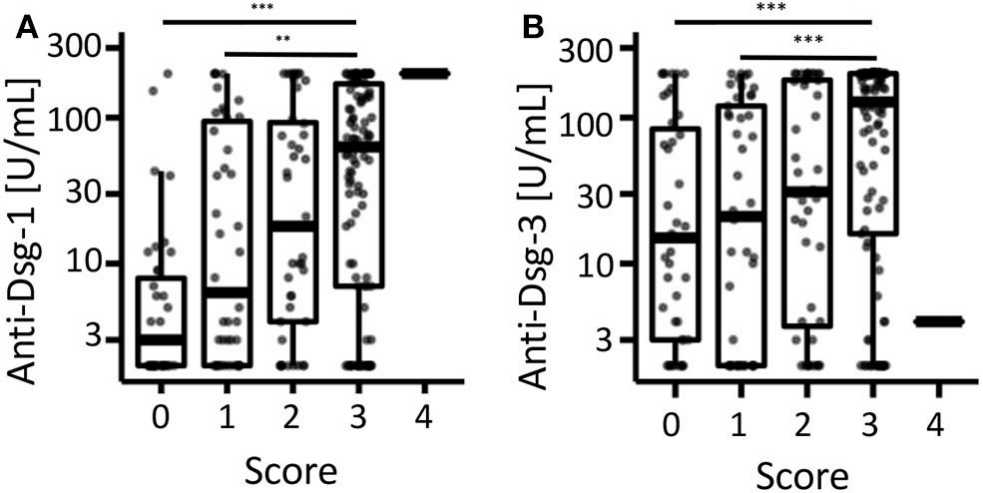
Correlation of anti-desmoglein-antibodies with the retrospective clinical score. The individual serum concentrations of **(A)** anti-desmoglein-1- and **(B)** anti-desmoglein-3-antibodies were depicted after stratification according to the retrospective score (0 = clear, 1 = almost clear, 2 = mild, 3 = moderate, 4 = severe). *** Indicates *P* < 0.001, Kruskal-Wallis-test with Dunn's *post hoc* test as *post hoc* and Holm's *p*-value adjustment.

**Figure 3:**
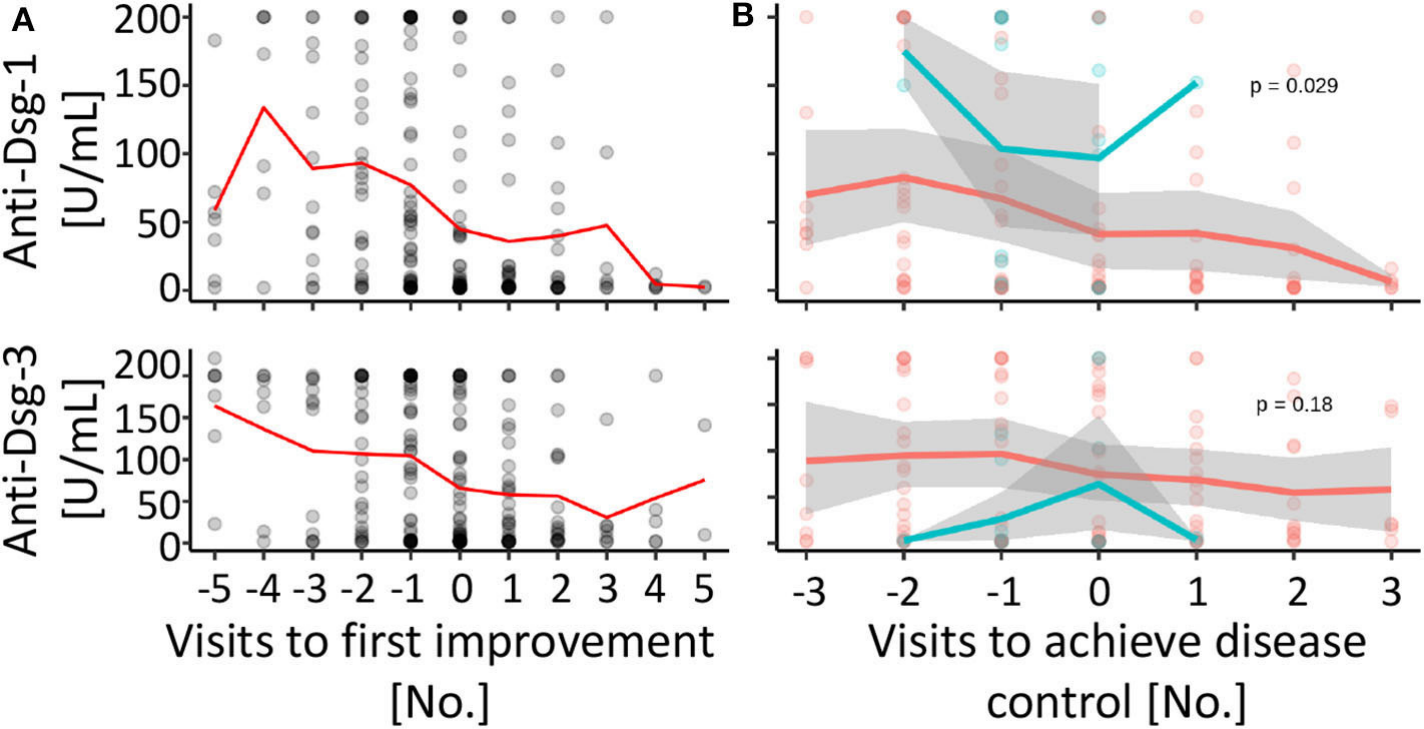
Anti-desmoglein serum antibodies decline over time, but are not associated with therapy changes. **(A)** The individual anti-desmoglein-1 and−3 serum concentrations (gray circles, overlapping values with a darker color intensity) and mean values (red line) were displayed for the respective visit [0 = first visit with clinical improvement, numbers of the visits before (negative) and after (positive) improvement]. **(B)** The autoantibody concentrations compared between subgroups with more (>4, red) or less (≤4, blue) than average therapy changes shown as individual values (circles) and mean (line) over time to achieve disease control (visit 0 = first visit with a severity score of 1 or 0). Gray area: 95% confidence interval. *P*-values calculated by repeated ANOVA.

Next, we investigated the association between anti-desmoglein-1 and −3 IgG serum concentrations and treatment changes. First, the data showed that the autoantibody concentrations were significantly elevated above a score of 2 regarding anti-desmoglein-1 (Figure 2A) and above a score of 3 regarding anti-desmoglein-3 (Figure 2B). In a small subpopulation of 4 therapy resistant patients the autoantibodies remained unaltered (11.5%, data not shown). Based on this finding, supporting a correlation between autoantibody concentrations and high disease activity, the serum autoantibody concentrations were analyzed over time and, focused on the visits before and after, clinical improvement was observed (Figure 3A). The data show declining mean values for both anti-desmoglein-1 and −3 antibodies between the first presentation and the last record (52–5 U/ml, *p* = 0.001, and 121–10 U/ml, *p* < 0.001, respectively). Of note, 15 of 66 patients with clinical remission expressed elevated autoantibody titers (23%, data not shown). To examine whether therapy changes are associated with the autoantibodies, the patient cohort was stratified into two subgroups according to the median therapy change, namely in few (≤4 changes) and many (>4 changes). The data showed that anti-desmoglein-1 serum concentrations, but not anti-desmoglein-3, differed between both subgroups (*p* = 0.029 and *p* = 0.18, Figure 3B). Of note, 15 of 66 patients (23%) who achieved disease control still had highly positive serology and 4 of 11 non-responders (36%) repeatedly exhibited high autoantibody levels (data not shown). Taken together, the autoantibody serum concentrations correlate with disease severity as such, but not with therapy changes.

## Discussion

To our knowledge, this is the first report considering therapy changes in pemphigus diseases as markers of a safe and effective therapy, i.e., few changes to achieve disease control. We determined an individual treatment response in the patients, which resulted from the comorbidities and disease activities and required an individual treatment sequence. No single treatment or sequence was identified to initiate disease control, except for rituximab courses. This may be due to the limited cohort size; however, it is more likely due to the heterogeneity of the presence of confounding factors that limit the use of the drugs and their versatile actions. These data suggest that in case of insufficient disease control, it may be better to add another drug than to replace the tolerated systemic drugs in order to achieve rapid disease control. That rituximab mediated disease control in 86% of the patients in this study underscores the central role of B cells in the pathogenesis of pemphigus disease and is in line with previous reports (1, 13–15). Thus, our data support the notion that rituximab is the most effective of all the available pemphigus treatments to induce disease control; however, severe pemphigus patients need to be carefully monitored.

The retrospective score used in this report reflected the requirements of treatment changes and was applicable from the data of a regular consultation documentation. We observed a correlation of the retrospective severity score with anti-desmoglein-1 and −3 autoantibody concentrations, in line with previous reports referring to other scoring systems (16–20). Moreover, we confirmed decreasing autoantibody concentrations during treatment, along with decreased disease activity. However, the use of validated scoring systems, such as the Autoimmune Bullous Skin Disorder Intensity Score (ABSIS) (21) or the Pemphigus Disease Area Index (PDAI) (7), could have identified more detailed differences than the 5-point retrospective scoring used here, but also require more documentation time for each patient visit. In future prospective studies, the use of validated scores will allow to generate comparability and meta-analysis. Overall, our data suggest that the anti-desmoglein antibody concentrations do not correlate directly with required future therapy changes. This may be due to the heterogeneity in disease endotypes, e.g., high autoantibody concentrations were still detectable in 23% of patients with disease control, and unaltered elevated autoantibody concentrations in a subgroup of therapy-resistant patients (11%). This supports the hypothesis that beyond the amount of autoantibodies the quality is also of importance, i.e., glycolization or sialylation (22), and also the type of antibody-secreting cell, i.e., rituximab-sensitive plasmablasts or therapy-resistant long-lived memory plasma cells (23).

Limitations: this analysis may be biased regarding the selection of treatments due to the monocentric setup. Moreover, separate documentation of oral and body lesions, damage, and inflammation and delineating the role of potential triggers may improve the data of future studies.

In summary, we observed that very good therapeutic control was achieved in most patients (66% disease control, 14% remission), but the therapy response was characterized by large individual variations, requiring several therapy changes. Predictive parameters are warranted in order to directly identify the optimal individual treatment regimen early after diagnosis, facilitating improvement of current treatment algorithms. The quantification of therapy changes may serve as a beneficial parameter to define safe and effective drugs for the treatment of pemphigus diseases.

## Data Availability Statement

All datasets generated for this study are included in the article/supplementary material.

## Ethics Statement

The studies involving human participants were reviewed and approved by Ethikkommission der Charité - Universitätsmedizin Berlin. Written informed consent for participation was not required for this study in accordance with the national legislation and the institutional requirements.

## Author Contributions

RS collected and analyzed the patients' data and wrote the manuscript. WF analyzed the data. MW wrote the manuscript. GH designed the project and wrote the manuscript. All authors contributed to the article and approved the submitted version.

## Conflict of Interest

RS has received a travel grant from ALK-Abelló Arzneimittel GmbH. MW has received speakers' honoraria for advisory boards and lecture activities from Mylan Germany GmbH, ALK-Abelló Arzneimittel GmbH, Allergopharma GmbH & Co. KG, HAL Allergie GmbH, Stallergenes GmbH, Bencard Allergie GmbH, DBV Technologies S.A, Novartis AG, Aimmune Therapeutics UK Limited, Sanofi-Aventis Deutschland GmbH, Regeneron Pharmaceuticals, Inc., Biotest AG, and Boehringer Ingelheim Pharma GmbH & Co. KG. GH performed consultancies for Allergopharma outside of this work and has lectured at educational events sponsored by Abbvie, Biotest, Eli-Lilly, and Sanofi. The remaining author declares that the research was conducted in the absence of any commercial or financial relationships that could be construed as a potential conflict of interest.
